# A changing picture of shigellosis in southern Vietnam: shifting species dominance, antimicrobial susceptibility and clinical presentation

**DOI:** 10.1186/1471-2334-9-204

**Published:** 2009-12-15

**Authors:** Ha Vinh, Nguyen Thi Khanh Nhu, Tran Vu Thieu Nga, Pham Thanh Duy, James I Campbell, Nguyen Van Minh Hoang, Maciej F Boni, Phan Vu Tra My, Christopher Parry, Tran Thi Thu Nga, Pham Van Minh, Cao Thu Thuy, To Song Diep, Le Thi Phuong, Mai Thu Chinh, Ha Thi Loan, Nguyen Thi Hong Tham, Mai Ngoc Lanh, Bui Li Mong, Vo Thi Cuc Anh, Phan Van Be Bay, Nguyen Van Vinh Chau, Jeremy Farrar, Stephen Baker

**Affiliations:** 1 The Hospital for Tropical Diseases, Ho Chi Minh City, Vietnam; 2 Oxford University Clinical Research Unit, Hospital for Tropical Diseases, Ho Chi Minh City, Vietnam; 3 Centre for Tropical Medicine, Nuffield Department of Clinical Medicine, Oxford University, Oxford, UK; 4 The MRC Centre for Genomics and Global Health, Oxford, UK; 5 Dong Thap Provincial Hospital, Dong Thap, Vietnam

## Abstract

**Background:**

Shigellosis remains considerable public health problem in some developing countries. The nature of *Shigellae *suggests that they are highly adaptable when placed under selective pressure in a human population. This is demonstrated by variation and fluctuations in serotypes and antimicrobial resistance profile of organisms circulating in differing setting in endemic locations. Antimicrobial resistance in the genus *Shigella *is a constant threat, with reports of organisms in Asia being resistant to multiple antimicrobials and new generation therapies.

**Methods:**

Here we compare microbiological, clinical and epidemiological data from patients with shigellosis over three different periods in southern Vietnam spanning14 years.

**Results:**

Our data demonstrates a shift in dominant infecting species (*S. flexneri *to *S. sonnei*) and resistance profile of the organisms circulating in southern Vietnam. We find that there was no significant variation in the syndromes associated with either *S. sonnei *or *S. flexneri*, yet the clinical features of the disease are more severe in later observations.

**Conclusions:**

Our findings show a change in clinical presentation of shigellosis in this setting, as the disease may be now more pronounced, this is concurrent with a change in antimicrobial resistance profile. These data highlight the socio-economic development of southern Vietnam and should guide future vaccine development and deployment strategies.

**Trial Registration:**

Current Controlled Trials ISRCTN55945881

## Background

Shigellosis is an ongoing global public health problem. Due to the fecal-oral transmission route of the organisms, the overwhelming burden of shigellosis is found in resource-poor settings with inadequate sanitation [1,2]. With an estimated number of episodes exceeding 90 million per annum in Asia alone, shigellosis represents a significant proportion of the total number of bacterial gastrointestinal infections worldwide [3]. Unlike other related bacteria which can cause a particular disease syndrome in specific locations (e.g. *Salmonella *Typhi) [4] it is a disease which "bridges the gap" between industrialized and developing countries. A report from the National Center for Infectious Diseases in the United States of America found the incidence of shigellosis to be 7.6 cases per 100,000 persons in 1993 [5].

The *Shigellae *are gram negative, non-motile bacilli of the larger bacterial family *Enterobacteriaceae. S. flexneri *are regarded to be the most abundant globally and are known to predominate in developing countries [3]. *S. sonnei *is the most commonly isolated species in developed countries, representing over 70% of the total isolates in the United States of America and Israel [5,6]. The disease syndrome associated with these organisms includes fever, headache, malaise, anorexia and occasionally vomiting, followed by excretion of profuse watery diarrhea proceeding bloody and/or mucoid diarrhea [7]. All the members of the genus *Shigella *are pathogens restricted to infecting humans and exert their effects on the gastrointestinal mucosa via the production of a multitude of virulence factors, including enterotoxins and effector proteins [8,9].

In a recent publication by von Seidlein *et al*. the authors found a change in dominant *Shigella *species related to the location in Asia (*S. sonnei *predominated in Thailand, *S. flexneri *was dominant in other Asian countries) and fluctuations in *S. flexneri *serotypes in the same location over the duration of the study [10]. The authors concluded that "*Shigella *appears to be more ubiquitous in Asian impoverished populations than previously thought and antibiotic-resistant strains of different species and serotypes have emerged" [10]. Such findings have important implications for treatment and prevention strategies of shigellosis.

On a larger scale, the *Shigellae *are a group of dynamic organisms, in which the overall bacterial population appears to be adaptable with a high recombination rate and a large amount of imported genetic material in the genome architecture [11]. These organisms are highly promiscuous regarding their ability to accept horizontally transferred genetic material. Like *E. coli *the *Shigellae *are successful recipients of numerous plasmids, which may be transferred from other enteric organisms in the gastrointestinal tract [12]. This is supported by evolutionary evidence that the *Shigellae *are a branch of the *E. coli *family, having developed a pathogenic phenotype by the acquisition of a virulence plasmid and other gene loci and genomic compensatory mechanisms [13,14].

It is known that the circulating species and serotypes may be considered a marker of the socio-economic climate in an individual setting [15]. It is clear that Vietnam has undergone rapid economic development since the early 1990's. To understand the nature of bacterial and clinical nature of shigellosis in southern Vietnam we have amassed and compared microbiological and epidemiological data on childhood shigellosis over three periods spanning 14 years, from 1995 to 2008.

## Methods

### Study sites and settings

The primary location was the pediatric gastrointestinal infections ward at the hospital for tropical diseases (HTD) in Ho Chi Minh City in southern Vietnam. The HTD is a 500 bed tertiary referral hospital treating patients from the surrounding provinces and from the districts within Ho Chi Minh City. The secondary location was Dong Thap provincial (DTP) hospital in Dong Thap province, approximately 120 km from the HTD in Ho Chi Minh City.

### Studies contributing data for analysis

Data from three independent studies were combined and compared. All patients enrolled in the three studies were treated as inpatients and there were no fatalities. The initial period (referred to as period A from here onwards) was a study performed at the pediatric ward at HTD from January 1995 to August 1996. The enrollment and clinical observations for this randomized controlled trial are as described previously [16]. Briefly, children that were aged >3 months and < l4 years, admitted to HTD with fever and bloody diarrhea (bloody diarrhea defined >3 loose stools with obvious blood) for <5 days were entered into the study provided that their parents or guardian gave fully informed consent. Additional strains for microbiological assessment only (nine in total) were collected for comparison within the same period of the study duration from DTP. Overall 80 strains were isolated from enrolled children over this period; clinical data was available for analysis on 63 patients with culture confirmed shigellosis.

The secondary period (referred to as period B from here onwards) was conducted only at the HTD, between March 2000 and December 2002. This period was a clinical and microbiological investigation of the etiology of diarrhea in the pediatric population admitted to the HTD in Ho Chi Minh City. Whilst the treatment criteria for this descriptive study were not controlled (> 90% of patients received treatment with fluroquinolones (norfloxacin or ofloxacin)), the remainder of the criteria for admission to the study were comparable, children were eligible for enrollment to the study if consent was given and they were aged less than 14 years. The obvious variation in the enrollment for this study was that children were enrolled on the basis of having any diarrheal syndrome, rather than specifically targeting those with dysentery and suspected shigellosis. One hundred and fourteen *Shigella *isolates were recovered during this period; clinical data was available for analysis on 113 patients.

The final period (referred to as period C from here onwards) in which data was combined was a trial conducted at the HTD and at DTP between June 2006 and December 2008. This was a randomized controlled trial for comparing the treatment of dysentery with ciprofloxacin and gatifloxacin in Vietnamese children (controlled trials number ISRCTN55945881) (HV and SB, *unpublished data*). The inclusion criteria were as period A. One hundred and three isolates were collected during this period and clinical data on all admitted children was available for analysis.

All three studies were approved ethical assessment by the Scientific and Ethical Committee of the hospital for tropical diseases and Oxford University tropical ethics committee (OXTREC) number 010-06 (2006).

### Microbiological methods

From all studies, stool samples were collected from patients and cultured directly on the day of sampling. Initial isolation was as below, however, all bacterial isolates were stored in glycerol at -80°C and re-serotyped and checked for consistency with the original antimicrobial susceptibility profile for the purposes of this work. All specimens were processed and checked in the microbiology laboratory of the HTD.

Samples were cultured overnight in selenite F broth (Oxoid, Basingstoke, UK) and onto MacConkey and XLD agar (Oxoid) at 37°C. Colonies suggestive of *Salmonella *or *Shigella *(non-lactose fermenting) were sub-cultured on to nutrient agar and were identified using a 'short set' of sugar fermentation reactions (Kliger iron agar, urea agar, citrate agar, SIM motility-indole media (Oxoid)). After incubation for 18 - 24 h at 37°C, the test media were read for characteristic *Shigella *reactions and API 20E test strips of biochemical reactions (Biomerieux, Paris, France) were used to confirm the identity of *Shigella spp*. Serologic identification was performed by slide agglutination with polyvalent somatic (O) antigen grouping sera, followed by testing with available monovalent antisera for specific serotype identification as per the manufacturers recommendations (Denka Seiken, Japan).

Antimicrobial susceptibility testing of all *Shigella *isolates against ampicillin (AMP), chloramphenicol (CHL), trimethoprim- sulfamethoxazole (SXT), tetracycline (TET), nalidixic acid (NAL), ofloxacin (OFX) and ceftriaxone (CRO) was performed by disk diffusion following standardized Clinical and Laboratory Standards Institute methods [17]. The minimum inhibitory concentrations (MICs) were additionally calculated for all isolates by E-test, according to manufacturer's recommendations (AB Biodisk, Solna, Sweden) and were compared to control strain *E. coli *ATCC 25922 and an in house fully sensitive *E. coli *control.

### Clinical observations and statistical analysis

Clinical data was recorded on specialized clinical report forms for all three studies by clinical staff involved in the studies. The data collected was related to basic details of the patient, age (months), sex, location of residence and weight (kg). A history from all patients was also recorded, including; duration of illness prior to admission to hospital (days), fever (defined as a prolonged temperature > 37.5°C), abdominal discomfort, vomiting, watery diarrhea (defined as three or more loose bowel movements during a 24-h period), bloody or mucoid diarrhea (defined as >3 loose stools with obvious blood or mucus), estimated number of episodes of diarrhea before attending hospital, convulsions believed to be related to fever and/or infection and if there was any known pretreatment with antimicrobials. A white blood cell count was performed on all patients and stools were examined by microscopy (HPF (× 400)) to identify white and red blood cells, these observations were scored on scale from zero to three, scale 0 = 0 cells/HPF, scale 1 = 1 to 10 cells/HPF, scale 2 = 11 to 20 cells/HPF and scale 3 = >20 cells/HPF. Time in hours (from initial investigation in hospital) to the ceasing of bloody/mucoid and watery diarrhea was recorded. Duration of hospital stay was recorded in days post admission; patients were only discharged when all clinical symptoms had resolved completely.

Data were double entered into Microsoft Excel for storage and manipulation. Mapping data was entered, analyzed and draw in MapInfo software (Pitney Bowes MapInfo Corporation, USA). For intergroup comparisons, Chi-square tests were used for comparison of categorical variables. For the analysis of continuous variables, Wilcoxon rank sum, and Kruskal-Wallis test were used for non-normally distributed data. A *p*-value of less than 0.05 (two-tailed) was considered significant. Statistical analysis was performed in R http://www.r-project.org/.

## Results

### Epidemiological findings

**Figure 1:**
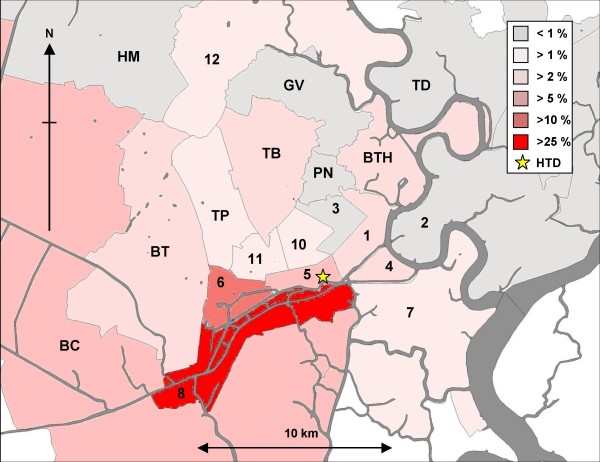
**The distribution of the residences of cases of childhood shigellosis admitted to the hospital for tropical diseases in Ho Chi Minh City**. Over the three periods we were able to positively identify infecting *Shigella *serotypes in the stools of 297 children with symptoms consistent with shigellosis. Of these patients, 228 (76.8%) children lived in the 23 districts that constitute Ho Chi Minh City. This figure represents the distribution of the homes of patients reporting to the hospital for tropical diseases with *Shigella *isolated from stool, by district. The percentage of cases reporting from each ward is distinguished by gradual shading. The location of the hospital for tropical diseases is shown by a yellow star. Large waterways (rivers and canals) are shown in dark grey shading.

Over the duration of the three periods spanning 14 years, 228 *Shigellae *were isolated from children living within 13 districts that constitute Ho Chi Minh City (Figure 1). Whilst the distribution of the location of the residences of these patients is biased by referral patterns and people attending the local hospital (HTD is one of several hospitals in the City where children may be treated for gastrointestinal infections), the majority of children attending HTD with culture confirmed shigellosis came from the three districts within the locality of the hospital (districts 5, 6 and 8), which constitutes a total population of over 800,000 people. In total, the majority of the patients resided in district eight (n = 88) within approximately 6 km of the hospital. There was no significant change in the locality of patients over the three periods, or any relationship between serotype and location of the residence of the patients.

**Figure 2:**
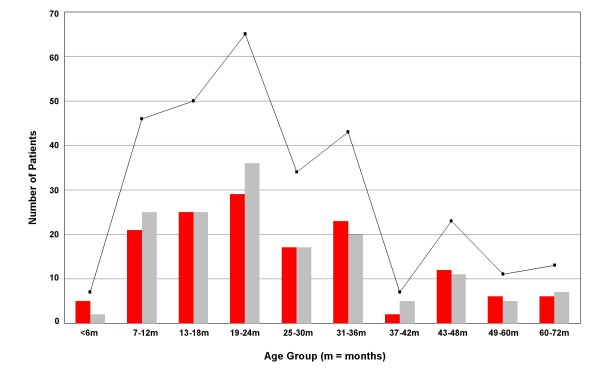
**The combined sex and age distribution of childhood shigellosis patients in southern Vietnam**. Graph depicts the combined age and sex distribution (female - red, male - grey) of 297 children with shigellosis. The black line with boxes represents the total number of cases per age group specified. The overall age range was from 3 months to 154 months, with a median of 24 months. There was no significant relationship of shigellosis with gender; in total, 152 patients were male (51.2%) and 145 were female (48.8%).

**Figure 3:**
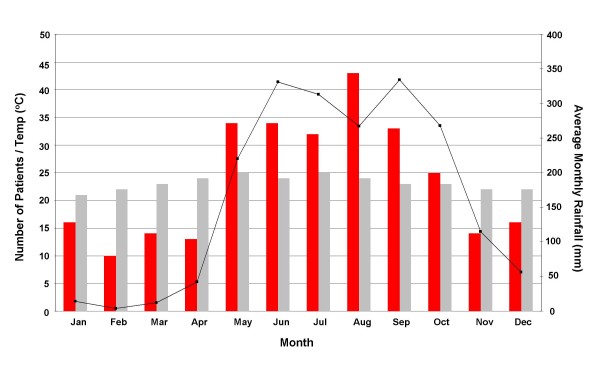
**The seasonal distribution of shigellosis in southern Vietnam**. southern Vietnam has two distinct seasons, wet and dry. The combined data were averaged by calculating the number of months represented to get an overall number of cases per month. Red bars; total number of cases, grey bars; average monthly temperature and black line with boxes; average monthly rainfall. The seasonal data represents the average rainfall and temperature per month for Ho Chi Minh City.

The median age of children with culture confirmed shigellosis from all the combined data was 24 months; the age range was from 3 to 154 months (Figure 2). The number of children requiring hospital treatment as inpatients for shigellosis declined significantly after 36 months of age. The combined data from periods A, B and C demonstrated some seasonality related to the times of peak infection, with the majority of cases (> 60%) occurring in the wet season (between May and September) (Figure 3).

### Microbiology and antimicrobial susceptibilities

**Figure 4:**
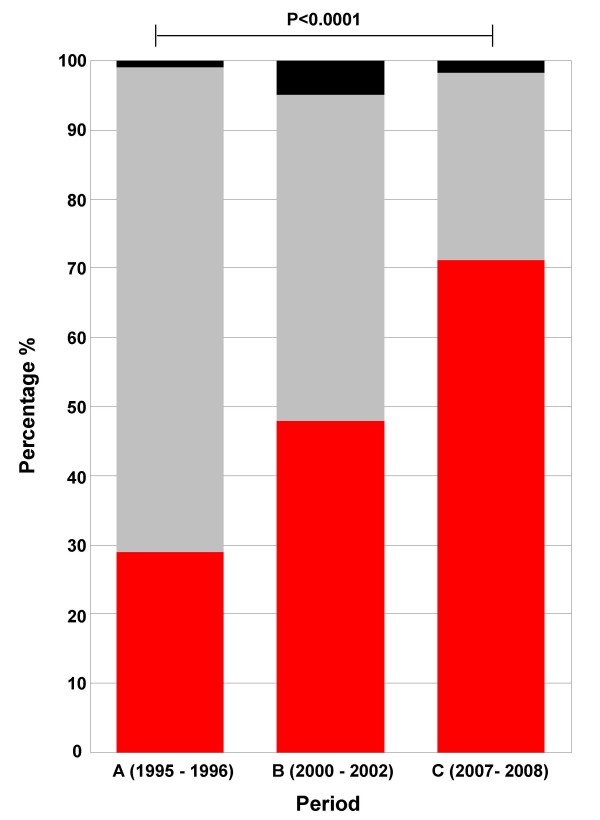
**The distribution of *Shigella *species from three childhood shigellosis studies in southern Vietnam over fourteen years**. The distribution of *Shigella *species from period A (n = 80), period B (n = 114) and period C (n = 103). The percentage of *S. sonnei *and *S. flexneri *are colored red and grey respectively, other *Shigella *species are colored black. The *p *value was calculated using the chi - squared test.

In total, 297 *Shigella *strains were isolated from periods A, B and C. Three were *S. boydii*, 136 were *S. flexneri*, 149 were *S. sonnei *and nine were untypeable. There was a significant species shift from *S. flexneri *to *S. sonnei *between period A (29% *S. sonnei*) and period C (78% *S. sonnei*) with an approximate 1:1 ratio of *S. flexneri *to *S. sonnei *in the intermediate period (Figure 4). Apart from *S. flexneri *serotype one only being found in period A, there was no evident fluctuations in *S. flexneri *populations between the three periods. The most commonly isolated *S. flexneri *serotype was serotype 2a; representing 43% of all the *S. flexneri *strains (Table 1).

**Table 1:** *Shigella flexneri *serotypes isolated in southern Vietnam between 1995 and 2008.

*S. flexneri *serotype	Number	Percentage (%)
1a	0	0
1b	0	0
1c	4	2.9
2a	59	43.4
2b	8	5.9
3a	13	9.6
3b	2	1.5
3c	16	11.8
4	7	5.1
4a	5	3.7
4b	1	0.7
4x	0	0
5a	0	0
6	13	9.6
x	0	0
y	0	0
Not typed	8	5.9

Total	136	100

**Figure 5:**
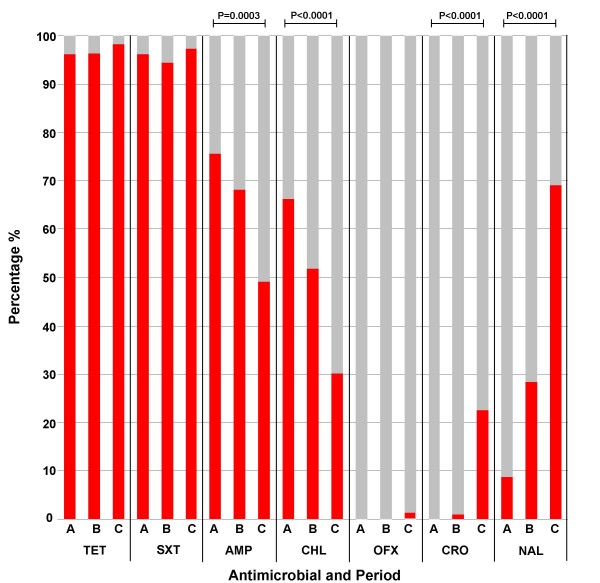
**Changing antimicrobial resistance patterns of *Shigella spp***. All organisms were tested for susceptibility to seven antimicrobial agents by the disc diffusion and E-test methods. The antimicrobials tested were as follows, AMP; Ampicillin, CHL; Chloramphenicol, SXT; Trimethoprim- Sulfamethoxazole, TET; Tetracycline, NAL; Nalidixic Acid, OFX; Ofloxacin and CRO; Ceftriaxone. Graph shows the percentage of resistant (red) and sensitive (grey) organisms isolated from periods A, B and C. Statistical significance was calculated using a chi squared test.

We identified a significant change in the profile of the proportions of organisms demonstrating resistance to seven antimicrobials (Figure 5). There was a sequential increase in the number of *Shigellae *isolated that were resistant to nalidixic acid, ofloxacin and ceftriaxone. In period C, 23% of strains were resistant to ceftriaxone and 68% were resistant to nalidixic acid (Figure 5). There was an additional overall increase in the number of antimicrobials to which the organisms were resistant. During period A, 62% of all *Shigellae *were resistant to three or more of the seven antimicrobials tested, this increased to 87% in period B and decreased to 83% in period C (Figure 5). The proportion of organisms that were resistant to trimethoprim- sulfamethoxazole and tetracycline was unchanged between the three periods (Figure 5). Between period A and C, there were significant decreases in the proportions of organisms resistant to ampicillin, decreasing from 75% to 48%, and chloramphenicol, decreasing from 66% to 30%.

**Figure 6:**
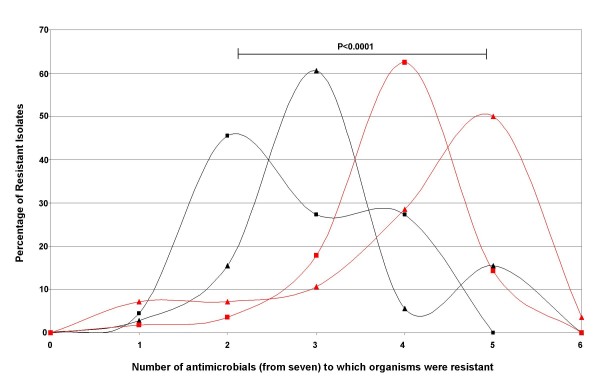
**The increasing proportions of antimicrobial resistant *S. sonnei *and *S. flexneri *during a fourteen year transition**. The distribution of the proportion of *S. sonnei *and *S*. *flexneri *isolates that were resistant to one or more of seven antimicrobials tested. *S flexneri *strains (red lines) were significantly more likely to be resistant to more antimicrobials that *S. sonnei *(black lines) over both collections compared. *S. sonnei *and *S. flexneri *were significantly more likely to be resistant to more antimicrobials when period C (2007 - 2008) (lines with triangles) was compared to period A (1995 - 1996) (lines with squares).

There was a discernible change in sensitivity patterns over time, which was also related to *Shigella *species (Table 2). *S. flexneri *was significantly more likely to be resistant to ampicillin in periods A and C and when combined over all three studies. *S. flexneri *was also significantly more likely to be resistant to chloramphenicol in periods B, C and overall (Table 2). The combined data demonstrated that *S. sonnei *was significantly more likely to be resistant to trimethoprim- sulfamethoxazole and ceftriaxone, despite ceftriaxone resistance not becoming evident till period C. The overall pattern of reversion of sensitivity to ampicillin and chloramphenicol was mainly observed with respect to *S. sonnei *isolates. An increase in the number of organisms resistant to multiple antimicrobials over time was seen in both *Shigella *species. However, between period A and period C, *S. flexneri *was more likely to be resistant to more antimicrobials than *S. sonnei *(Figure 6). Resistance to multiple antimicrobials increased from two to three out of the seven tested from periods A to C for *S. sonnei *and from four to five from the seven antimicrobials tested from periods A to C for *S. flexneri *(Figure 6).

**Table 2:** Comparison of resistance patterns between *Shigella flexneri *and *Shigella sonnei *isolated in southern Vietnam between 1995 and 2008.

Collection	Serotype (n)	Phenotype^b^	AMP	CHL	SXT	TET	NAL	OFX	CRO
A (1995 - 1996)	*sonnei *(24)	R	11	8	23	23	0	0	0
		S	13	16	1	1	24	24	24
	*flexneri *(56)	R	54	10	53	53	9	0	0
		S	2	46	3	3	47	56	56
			
*p *Value^a^			< 0.0001	0.1287	0.8102	0.8102	0.0371	-	-

B (2000 - 2002)	*sonnei *(54)	R	50	10	53	52	9	0	1
		S	4	44	1	2	45	54	53
	*flexneri *(50)	R	46	38	44	49	21	0	0
		S	4	12	6	1	29	50	50
			
*p *Value^a^			0.9316	< 0.0001	0.0415	0.5577	0.0052	-	0.329

C (2007 - 2008)	*sonnei *(71)	R	17	5	71	69	51	0	12
		S	54	66	0	2	20	71	59
	*flexneri *(30)	R	25	28	28	30	19	1	1
		S	5	2	2	0	11	29	29
*p *Value^a^			< 0.0001	< 0.0001	< 0.0001	0.3696	0.4619	0.297	0.076

Combined	*sonnei *(148)	R	78	23	147	144	60	0	13
		S	70	125	1	4	88	148	135
	*flexneri *(136)	R	125	76	125	132	49	1	1
		S	11	60	11	4	87	135	135
			
*p *Value^a^			< 0.0001	< 0.0001	0.001	0.613	0.365	0.478	0.002

^a ^*p *Value calculated using the chi-squared test

^b ^Phenotype with respect to Resistant (R) or Sensitive (S)

AMP; Ampicillin, CHL; Chloramphenicol, SXT; Trimethoprim- Sulfamethoxazole, TET; Tetracycline, NAL; Nalidixic Acid, OFX; Ofloxacin, CRO; Ceftriaxone.

### Clinical features associated with shigellosis

Clinical data was combined and analyzed from all three studies; this permitted a comparison of some of the features of the patients with confirmed shigellosis over the three studies. Data were available for analysis from 279 patients; 63 patients from period A, 113 patients from period B and 103 patients from period C (Table 3). These data demonstrated several changes in disease profile over the three periods. There was a statistically significant increase in age, which corresponded with an increase in weight of the children from period A to period C (Table 3). There was decrease in the number of days of history of the disease symptoms prior to admission to hospital. There was a statistically significant increase in the number of children with watery diarrhea, abdominal pain and febrile convulsions. These clinical features combined suggested a progressively more severe infection syndrome between 1995 and 2008. Additionally, patients in period C, had higher white blood cell counts. Over the 14 year period, patients had a higher density of white cells in their stool and had longer stays in hospital.

**Table 3:** Clinical results of *Shigella *infections between 1995 and 2008.

	A (1995 - 1996)	B (2000 - 2002)	C (2007 - 2008)	Combined	*p *value^a^
**Pateints**	*n *= 63	*n *= 113	*n *= 103	*n *= 279	

Age (months)^d^	23 (17 - 48)	21 (14 - 29)	30 (19 - 42)	24 (16 - 36)	< 0.001
Weight (kg)	10 (9 - 13)	10 (9 - 12)	11.5 (10 - 15)	10.5 (9 - 13)	0.004
Male sex (%)	31 (49)	50 (44)	61 (59)	184 (59%)	0.085
					
**Patient history**					

Days	2 (1 - 7)	2 (1 - 9)	1 (1 - 4)	2 (1 - 9)	< 0.001
Fever (%)	62 (98)	104 (92)	100 (97)	266 (95)	0.09
Abdominal pain (%)	33 (52)	41 (36)	79 (76)	153 (54)	< 0.001
Vomitting (%)	24 (38)	64 (56)	51 (49)	139 (50)	0.062
Watery diarrhea (%)	28 (44)	67 (59)	74 (71)	169 (60)	0.002
Bloody/mucoid diarrhea (%)	63 (100)	60 (53)	98 (95)	221 (79)	< 0.001
Diahorreal episodes per day	NA	8 (5-10)	8 (5-10)	8 (5-10)	0.595
Convulsions (%)	4 (6)	7 (6)	20 (19.4)	31 (11)	< 0.001
Known pretreatment (%)	3 (5)	8 (7)	4 (4)	14 (5)	0.543
					
**Clinical details**					

Serotype *sonnei *(%)	21/63 (33)	55/113 (49)	71/103 (69)	153/279 (55)	< 0.001
White cell count (× 10^3^/mm^3^)	10 (8.3 - 15)	10.1 (7.7 - 12.8)	13.1 (10.1 - 17.3)	11.3 (8.7 - 15.4)	< 0.001
Red cells in stool^b^	NA	1	1	1	0.715
White cells in stool^b^	NA	3	3	3	0.02
Mucoid duration (hrs)	31.5 (24 - 53.5)	36 (24 - 54)	28 (18 - 48)	30 (19.5 - 48)	0.113
Diarrhea duration (hrs)	48.5 (29.25 - 87)	48 (24 - 72)	48 (30 - 72)	48 (26.75 - 72)	0.402
					
**Duration of illness**					

Hospital stay (days)	3 (1 - 12)	4 (1 -15)	5 (2 - 14)	4 (1 -15)	< 0.001
Disease duration (days)^c^	4 (2 - 15)	6 (3 - 18)	6 (3 - 15)	6 (2 - 18)	< 0.001

^a ^*p *Values calculated using either Chi-square test or the Kruskal-Wallis test

^b ^Cells in Stools assessed as described in methods

^c ^Disease duration calculated by addition of history of disease and stay in hospital

^d ^Interquartile range values in brackets unless stated

The increase in the severity of the disease was concurrent with a change in antimicrobial resistance profiles of the organisms and a change in the dominant *Shigella *species isolated. Therefore, these data suggested a more severe disease pattern may be related to infection with *S. sonnei*. To account for any variation in disease syndrome that may be species specific, the data were analyzed to compare the clinical syndromes related to species. The data presented in Table 4 demonstrates only subtle differences between the syndromes synonymous with the two differing species. *S. flexneri *shows an increase in the number of days of illness prior to admission in hospital, the number of episodes of diarrhea, an increase in the duration of mucoid/bloody diarrhea and the duration of stay in hospital.

**Table 4:** The clinical presentation of *Shigella flexneri *and *Shigella sonnei *infections.

	*S. flexneri*	*S. sonnei*	*p *value^a^
**Pateints**	*n *= 123	*n *= 147	

Age (months)^d^	25 (12 - 42)	23 (14 - 36)	0.105
Weight (kg)	11 (8.5 - 14)	10 (9.9 -13)	0.558
Male sex (%)	55 (44.7)	83 (56.5)	0.055

**Patient history**			
			
Days	2 (2 - 3)	1 (1 - 2)	< 0.001
Fever (%)	117 (95)	141 (96)	0.761
Abdominal pain (%)	64 (52)	84 (57.1)	0.48
Vomitting (%)	60 (48.8)	74 (50.3)	0.78
Watery diarrhea (%)	78 (63.4)	86 (58.5)	0.41
Bloody/mucoid diarrhea (%)	97 (78.9)	117 (80)	0.88
Diarrhea episodes per day	10 (5 - 10)	8 (5 - 10)	0.051
Convulsions (%)	9 (7.3)	21 (14.3)	0.07
Known pretreatment (%)	7 (5.7)	7 (4.8)	0.585
			
**Clinical details**			

White cell count (× 10^3^/mm^3^)	10 (8 - 13.6)	12 (10.5 - 15.5)	0.029
Red cells in stool^b^	1	1	0.056
White cells in stool^b^	3	3	0.173
Mucoid duration (hrs)	36 (24 - 53.5)	25 (18 - 48)	0.054
Diarrhea duration (hrs)	48 (39 - 72)	48 (27 - 72)	0.088
			
**Duration of illness**			

Hospital stay (days)	5 (4 - 5)	4 (3 - 5)	0.276
Disease duration (days)^c^	7 (6 - 8)	5 (4 - 7)	0.009

^a ^*p *Values calculated using either the Chi-square test or the Kruskal-Wallis test

^b ^Cells in Stool assessed as described in methods

^c ^Disease duration calculated by addition of history of disease and stay in hospital

^d ^Interquartile range values in brackets unless stated

## Discussion

Our findings demonstrate that the epidemiology of shigellosis infection is similar in southern Vietnam to other locations in Asia. The main burden of infection in children is in those under three years of age [10,15,18,19]. The median age of patients in this investigation was 24 months, this is slightly less than a previous study in Nha Trang, Central Vietnam [10]. A discrepancy in age in the two settings may be related to the epidemiological study being performed with ongoing community surveillance, rather than those admitted to hospital for treatment. We also found a pattern of infection which correlated with the rainy season. The observation that *Shigella *infections generally coincide with the wet season in a tropical setting has been noted before in an urban setting in Jakarta, Indonesia [18]. Transmission of *Shigella *has been associated with wastewater and river water in Vietnam in two independent locations in Vietnam [20,21]. An increase in fecal contamination of the water supply due to increased ground water may account for this pattern as distance to a water source was found to be associated with higher risk of shigellosis in Nha Trang. The majority of patients enrolled in the studies combined here resided in District 8 of Ho Chi Minh City. Although we are unable to draw meaningful conclusions from the residences of these patients owning to referral and catchment areas of the HTD, district 8 represents the area of the city with the greatest density of canal networks and waterways.

In addition to a species shift over time, there was combined effect on antimicrobial resistance; there was a marked increase in resistance to ceftriaxone and nalidixic acid. We have previously reported an alarming increase in ceftriaxone resistant *Shigellae *in southern Vietnam [22]. Whilst nalidixic acid is no longer used therapeutically, resistance increases the MIC to fluoroquinolones, which are recommended for the treatment of *Shigella *infections [23]. Our theory that antimicrobial resistant organisms are under selective pressure in this population is supported by a sequential decrease in resistance to older antimicrobial therapies, such as ampicillin and chloramphenicol which are now rarely used in the community to treat gastrointestinal infections. The uncontrolled use of antimicrobials in this setting may fuel the spread of multiple drug resistant organisms. However, due to promiscuous nature of the *Shigellae *it is likely that resistance genes are transferred regularly to and from other enteric bacteria and maintained by selective pressure. The change is species and antimicrobial resistance pattern reflects a change occurring in the *Shigella *population over time in this setting. Locality and time of isolation data suggest that entrance to all studies was sporadic and there was no evidence of transient epidemics.

Currently there are several candidate *Shigella *vaccines in development, of which some have already been tested in initial clinical trials [24-27]. The development and deployment of *Shigella *vaccines may be hindered by the number of different species and serotypes circulating in one setting and in differing locations. For example, *S. flexneri *serotypes are known to fluctuate over time, this has been observed in India, Indonesia, Bangladesh, and Pakistan, [10,28]. Here, we have demonstrated a significant longitudinal transition of species from *S. flexneri *to *S. sonnei*. Vaccine development for shigellosis is challenging as primary infection offers only serotype specific immunity [29]. A study concerning a cohort of Chilean children found infection conferred 76% protective efficacy against re-infection with the same serotype [30]. An option for controlling shigellosis would be the development of a series of single serotype vaccines which could be implemented in individual locations with a known serotype profile. Alternatively, the most cost effective method of control would be the development of a polyvalent vaccine offering cross protection to a number of known dominant serotypes, this approach may aid in tackling the global burden of shigellosis. The transition of dominant *Shigella *species in southern Vietnam has occurred on a background of economic development and may predict a continuing cycle in other areas under going similar rapid economic changes.

## Conclusions

What we are unable to specifically ascertain from this study is the overall incidence and greater epidemiological picture of shigellosis in this setting. On the basis of these data a thorough epidemiological assessment of burden is warranted to calculate the financial and health implications of any potential future routine vaccination against shigellosis that may become available. However, here we have shown a significant transition in *Shigella *species and antimicrobial resistance dominance overtime and a concurrent change in the clinical disease presentation.

## Competing interests

The authors declare that they have no competing interests.

## Authors' contributions

NTKN, TVTN, PHD, JIC, NVMH, TTTN, PVM, CTT, PVBB and TSD performed the microbiological culturing, sensitivity testing and serotyping. MFB, PVTM provided critical analysis related to this work. HV, CP, LTP, MNL, BLM, VTCA, PVBB, HTL, MTC, NTHT, NVVC and JF conducted the clinical work providing the data for analysis. HV, JF, MFB and SB conceived the study, analyzed and interpreted the data and prepared the manuscript. All authors have read and approved the final version of this manuscript.

## Pre-publication history

The pre-publication history for this paper can be accessed here:

http://www.biomedcentral.com/1471-2334/9/204/prepub
